# Preparation and Characterization of Optically Active Polyurethane from Rotatory Binaphthol Monomer and Polyurethane Prepolymer

**DOI:** 10.3390/molecules26102986

**Published:** 2021-05-18

**Authors:** Ling Lin, Haiyan Mao, Ziyin Li, Wenyao Li, Chaoxia Wang

**Affiliations:** 1 Key Laboratory of Eco-Textile, School of Textiles and Clothing, Jiangnan University, Ministry of Education, 1800 Lihu Road, Wuxi 214122, China; janegirl007@163.com; 2 Yancheng Institute of Technology, College of Textiles and Clothing, Yancheng 224051, China; mao-hai-yan@hotmail.com (H.M.); lzyinann@163.com (Z.L.); 3 School of Materials Engineering, Shanghai University of Engineering Science, Shanghai 201620, China; liwenyao314@gmail.com

**Keywords:** optically active polyurethane, structure, thermal insulation, infrared emissivity

## Abstract

Optically active polymers are promising multifunctional materials with great application potentials. Herein, environmentally friendly optically active polyurethanes (OPUs) were obtained by introducing rotatory binaphthol monomer to polyurethane. The influence of binaphthol monomer content on the structure, mechanical properties, infrared emissivity, and thermal insulation of OPUs was studied intensively. Structure characterization indicated that the optically active polyurethanes have been successfully synthesized. The OPU synthesized with BIMOL and BDO at the mole ratio of 1:1 presented better thermal resistance. In addition, OPUs showed enhanced tensile strength and stretchability with the increase of BINOL content to a certain extent due to its rigid structural features and high molecular weight. The optically active polyurethanes showed lower infrared emissivity values (8–14 μm) than waterborne polyurethane (WPU), and the infrared emissivity decreased from 0.850 to 0.572 as the content of the BINOL monomer increased. Moreover, OPU4 exhibited the best heat insulation and cooling ability with about a 7 °C temperature difference. The thus-synthesized optically active polyurethanes provide an effective solution for developing highly effective thermal insulation materials.

## 1. Introduction

The human body is subject to constantly changing environmental conditions varying from midsummer to severe winter, resulting in uncomfortable feelings that affect the well-being of people [1,2]. Textiles and buildings are the main gears to protect humans from hazards from external conditions and to maintain the well-being of people. However, common textiles or buildings are not capable enough of protecting humans, especially in some extreme circumstances such as extreme weather conditions. Thermal insulation materials with superior heat blocking or insulation capacity are thus highly demanded in developing protective textiles and buildings [3,4,5]. These thermal insulation materials are capable of reflecting and/or absorbing heat, buffering, or preventing the free heat exchange between people and the external environment. The interior temperature of humans and the indoor temperature of buildings are thus maintained at a comfortable level appropriate for human well-being and without having to run air conditioning [6,7,8].

Waterborne polyurethanes (WPUs) are promising polymeric materials due to the advantage of environmental friendliness, good thermal and chemical stability, high flexibility, and structural adjustability [9]. They have been widely applied in textiles, buildings, stealth materials, optical devices, etc. In heat insulation, WPUs usually act as adhesives and coatings owing to their excellent film-forming capacity. They are always mixed with nanoparticles (TiO_2_, ZnO, cenosphere, etc.) to capture these particles for preparing thermal insulation coatings. For example, Lu et al. [10] prepared thermal insulation coatings with antimony doped nano-SnO_2_/waterborne polyurethane composite emulsion. Due to the absorbance of the infrared light by the introduction of antimony into nano-SnO_2_ particles, the surface temperature of the coated glass increased while the temperature inside decreased about 17.5 °C under the illumination of sunlight. Zhu et al. [11] used Mg(OH)_2_ particles as a function reagent and polyurethane as a binder to prepare coating adhesive for finishing cotton fabrics. The cotton fabrics showed the best thermal insulation effect with 0.12 mm coating thickness. Xu et al. [12] prepared ATO nanoparticles modified WPU emulsion for coating glass slides. The glass coated with ATO/WPU films could effectively prevent heat diffusion and heat transmission with an enhanced thermal insulating effect. However, the added polyurethanes usually deteriorated the thermal barrier effect of nanoparticles, which has greatly confined the application potential of the thus-developed thermal insulation materials. Therefore, it is important to develop a type of polyurethane that possesses reasonable intrinsic thermal insulation properties, which can maintain the excellent thermal insulation performance of nanoparticles or even achieve better thermal insulation performance.

Recently, increasing attention in research has been put on optically active polymers for their orderly secondary structure, adjustable chiral parameter, abundant interchain interaction, and other unique features [13,14,15]. These optically active polymers have been widely used in chiral recognition, enantiomeric resolution, nonlinear optics, biomedicine, liquid crystal, optical switches, etc. [16,17]. Currently, optically active polymers mainly include acrylates, polyesters, polyamides, polyimides, and maleimides, and polyurethanes. Among them, optically active polyurethanes are mainly prepared from natural sugars, amino acids, tartaric acids, chiral binaphthyl groups, artificially synthesized optically active diols or polyols, and diisocyanates by addition polymerization [18,19]. Chiral binaphthyl groups with torsional, noncoplanar special rigid structures are preferred chiral sources for the preparation of these optically active polymers [20]. For example, Xi et al. [21] prepared three optically active cyclic and linear poly(aryl esters) from chiral 1,1-bi-2-naphthol reacting with terephthaloyl, isophthaloyl, and o-phthaloyl chloride via condensation reactions. The introduction of the binaphthyl groups into the polyurethane chain is simple but effective in harnessing optically active properties. Some investigations have been carried out to study the infrared emissivity of optically active polyurethanes. Chen et al. [22] used chiral 1,10-binaphthyl and 2,4-toluene diisocyanate to synthesize optically active polyurethanes, and the obtained polyurethane exhibited larger optical rotation than binaphthyl monomer and stronger CD signals with positive and negative cotton effect. After the optically active polyurethanes were mixed with TiO_2_, the obtained nanocomposites possessed a lower emissivity. Nakano et al. [23] reported a cyclic dimer and a cyclic trimer that were prepared with (R)-1,1′-Bi(2-naphthol) and 1,4-phenylene diisocyanate. The polymer showed a rather stiff, π-stacked and 2/1-helical conformation. However, most investigations about the optically active polyurethanes were focused on lowering infrared emissivity, while their applications as thermal insulators are seldom investigated.

In this work, environmentally friendly optically active polyurethanes were successfully fabricated via introducing rotatory binaphthol monomer into polyurethane prepolymer that was synthesized with IPDI and PCDL. The effect of binaphthol monomer content on the thermal stability, mechanical properties, infrared emissivity, and thermal insulation of obtained optically active polyurethanes were measured and discussed in detail. The synthesized optically active polyurethane showed tunable mechanical properties and infrared emissivity, together with a heat-shielding performance, by varying the content of the BINOL monomer. The thus-synthesized OPU provides a new strategy for developing advanced thermal insulation materials for protective clothing and building.

## 2. Results and Discussion

### 2.1. FTIR Spectra

**Figure 1:**
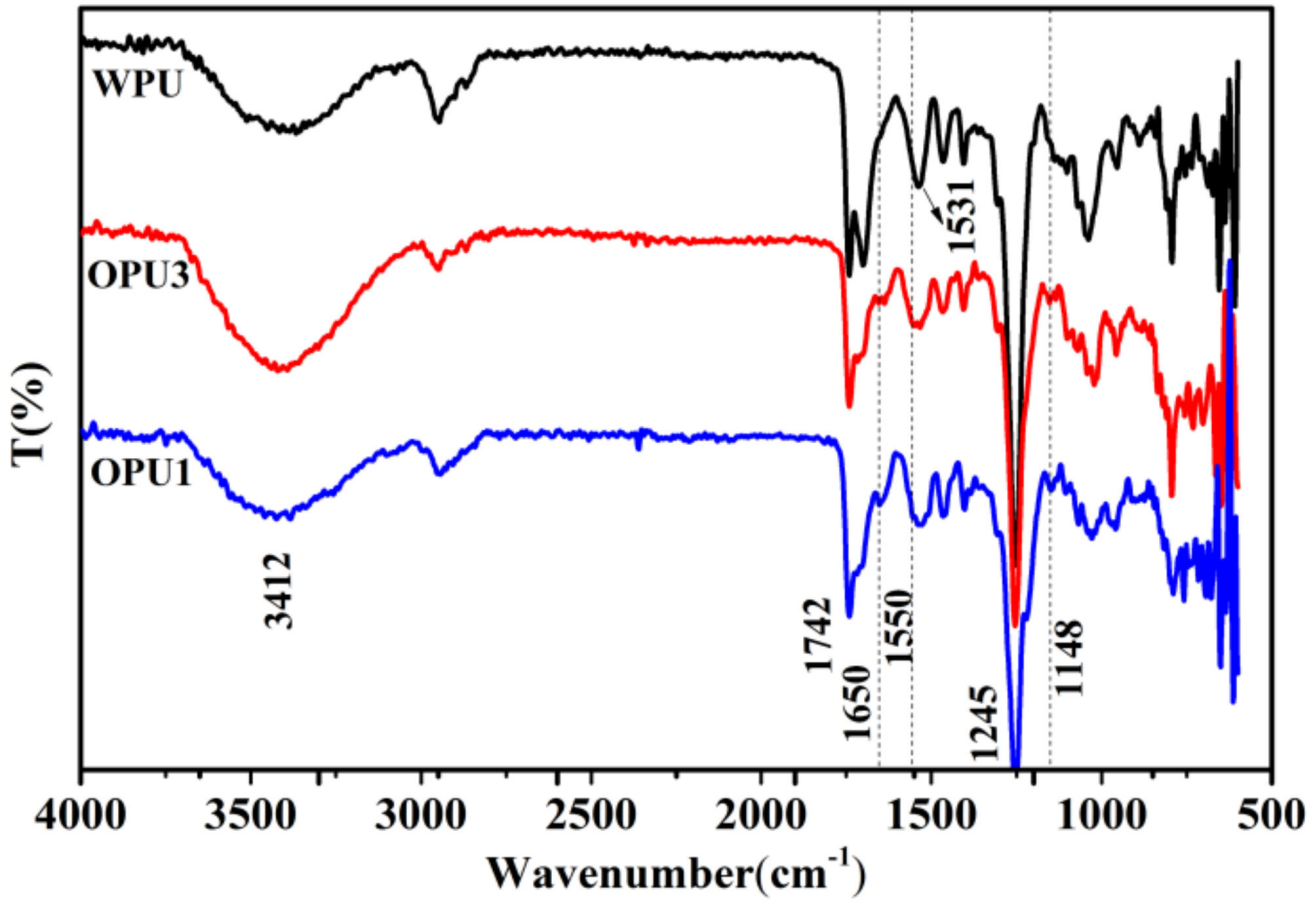
FTIR spectra of WPU and OPUs.

Representative FTIR spectra of OPUs are presented in Figure 1. The typical absorption peaks at 3412 cm^−1^, 1742 cm^−1,^ and 1245 cm^−1^ are related to the stretching vibration of N-H, C=O, and C-O in the urethane groups of polyurethane. The N-H bending and the C-N stretching vibrations in the urethane groups are also observed near 1531 cm^−1^. Moreover, no obvious absorption peak around 2270 cm^−1^ ascribed to the isocyanate group (-NCO) has been detected, indicating the complete reaction of -NCO and –OH groups. All these peaks demonstrated the formation of polyurethane chains.

Compared with WPU, a new peak appears at 1650 cm^−1^ for OPU, which is assigned to the stretching vibration of C=O in the urethane groups formed by the reaction of IPDI and BINOL. In addition, the characteristic peaks corresponding to the benzene ring can be observed at 1148 cm^−1^ in OPU1 and OPU3, indicating that BINOL has been successfully incorporated into the polyurethane chain. The FTIR spectra have confirmed the successful synthesis of optically active polyurethane.

### 2.2. H NMR Spectra

**Figure 2:**
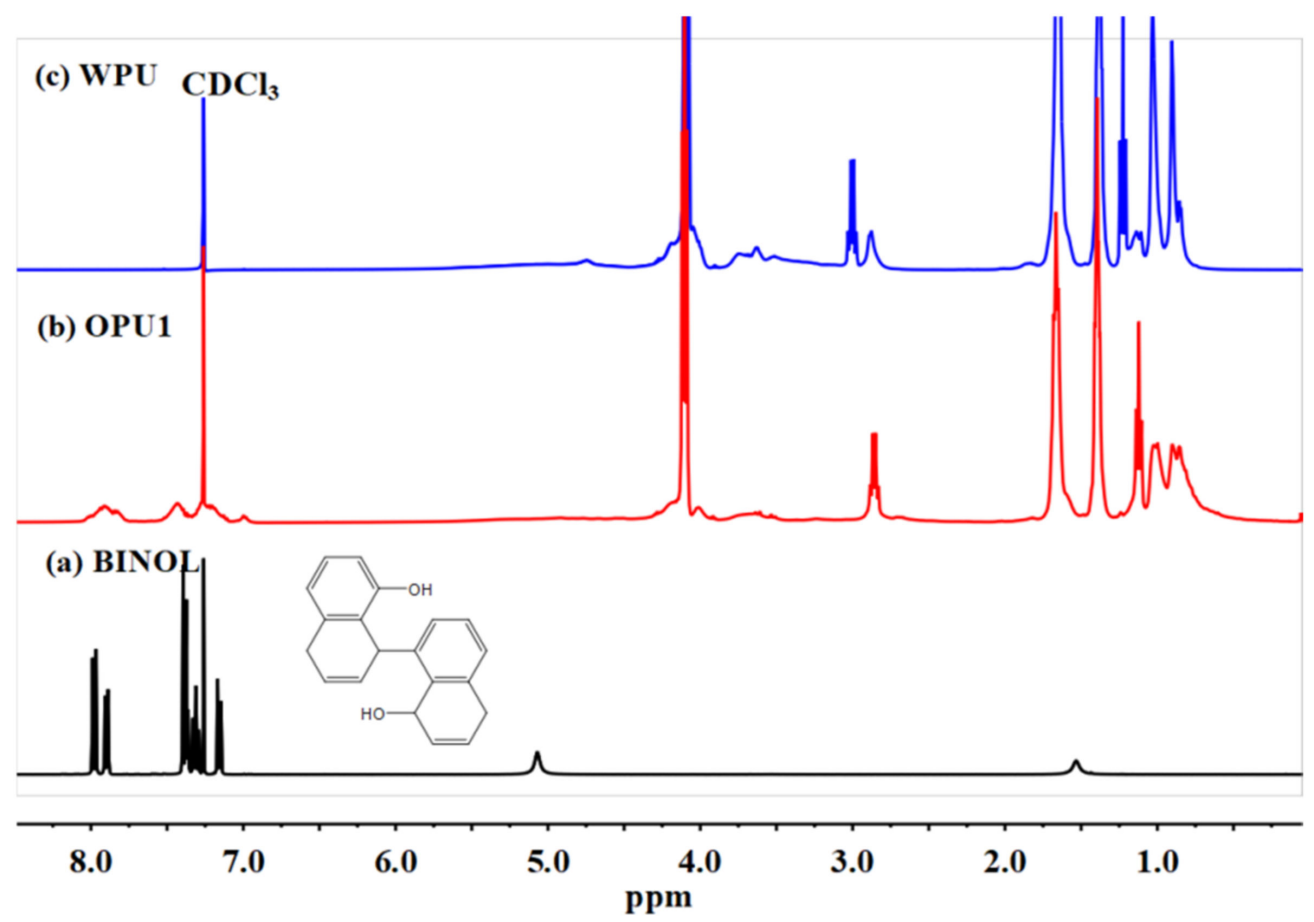
1H NMR spectra of BINOL, OPU1, and WPU: (**a**) BINOL, (**b**) OPU1, (**c**) WPU.

^1^H NMR spectra were used to further confirm the structure of OPUs, as shown in Figure 2. The chemical shifts of BINOL assigned to the protons in the naphthalene ring appear at 7.11–7.99 ppm, and the peaks at 5.07 ppm and 1.53 ppm belong to the protons in phenolic hydroxyl groups. As for WPU and OPU1, the peaks at 4.07–4.11 ppm and 4.01 ppm are ascribed to the CH_2_CH_2_OCOO– protons in PCDL soft segment and the CH_3_C(COOH)(CH_2_O)_2_– protons in DMPA, respectively. The chemical shifts at 2.88–3.03 ppm are related to the CH_2_NHCOO- protons in IPDI. Signals between 0.85 and 1.67 ppm mainly belong to the CH_2_C(CH_2_)_2_CH_2_C or OCH_2_CH_2_CH_2_CH^2^O protons in IPDI and BDO. In addition, compared with the NMR spectrum of WPU, the signals ascribed to the protons in the naphthalene ring in OPU1 are observed at 7.1–8.0 ppm, demonstrating the existence of BINOL in the OPU1 structure. It is thus evident that optically active polyurethane has been successfully synthesized as the existence of BINOL in OPUs can be detected.

### 2.3. Molecular Weight

The molecular weight of OPUs was investigated by GPC, as shown in Table 1. It is evident that the number-averaged molecular weight (Mn) of OPUs is in the range of 15,960–19,444 g/mol. The polydispersity index (PDI) is about 1.76–2.62, indicating a relatively narrow molecular weight distribution. In addition, the molecular weight of OPU1 prepared with BINOL is lower than OPU3 and WPU, and this is probably because the reactivity of phenolic hydroxyl groups in BINOL is relatively lower than that of alcoholic hydroxyl groups in BDO.

### 2.4. Thermal Stability

**Figure 3:**
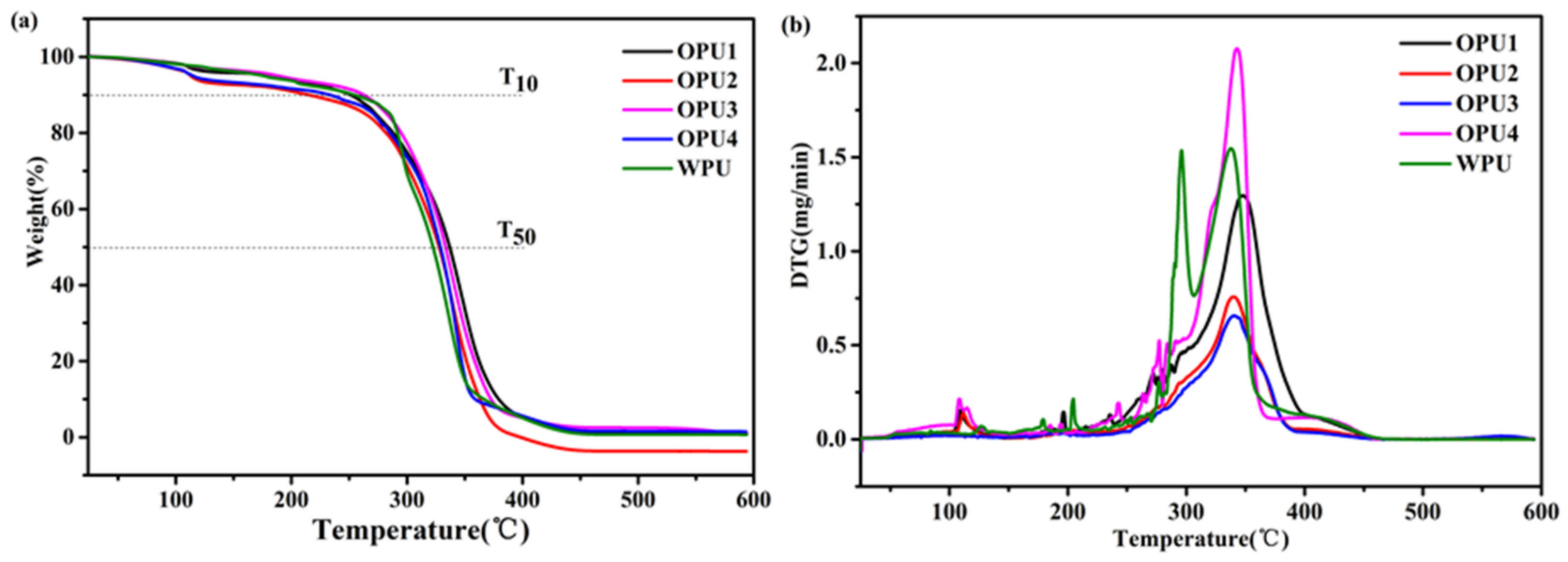
Thermal stability of WPU and OPUs: (**a**) TGA curves and (**b**) DTG curves.

The thermal stability of OPUs was characterized by TGA and DTG curves, as shown in Figure 3. In Figure 3a, the slight weight loss at around 110 °C is due to the residual water extraction as a result of heating. The main weight loss occurs in the range of 250–310 °C and 310–460 °C, corresponding to the decomposition of urethane bonds and the soft segments, respectively. In Figure 3b, WPU shows a distinct two-step weight loss, while OPUs presents onset weight loss. The decomposition temperatures of 10% weight loss (T10) of OPU3 and WPU are similar, which are slightly higher than other samples. In addition, the decomposition temperatures of 50% weight loss (T50) of OPU1 (337 °C) and OPU3 (333 °C) are enhanced by 5–15 °C, compared to WPU (322 °C), OPU2 (326 °C), and OPU4 (328 °C). Therefore, the above results indicate that OPU3 synthesized with BIMOL and BDO at mole ratio 1:1 presents better thermal stability.

### 2.5. XRD Pattern

**Figure 4:**
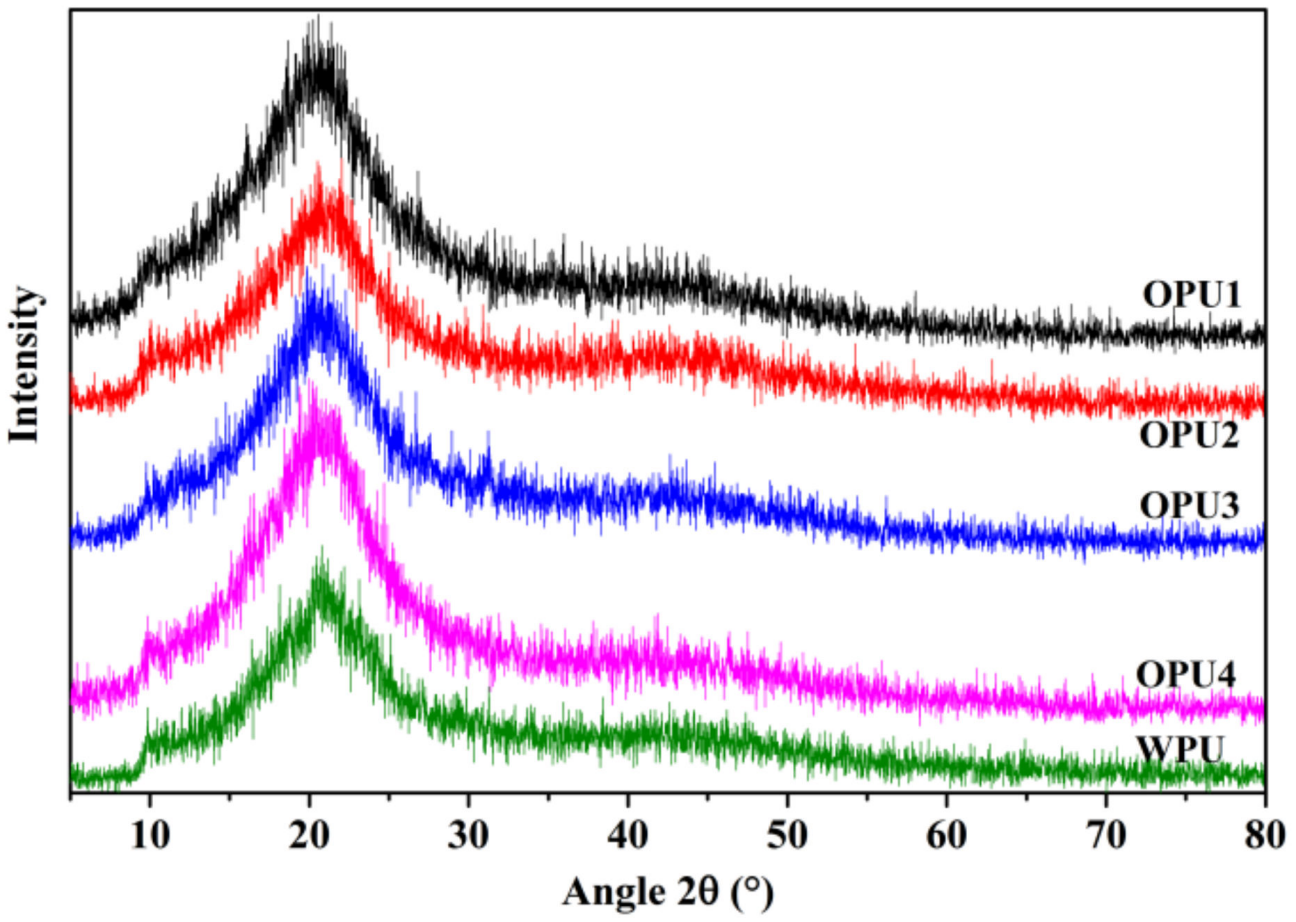
XRD patterns of WPU and OPUs.

The XRD patterns were obtained to detect the structural feature of OPUs, as depicted in Figure 4. The broad characteristic peak around 20.8° for both WPU and OPUs indicates the amorphous structure and nonanisotropic behavior of the synthesized polyurethanes. Overall, both WPU and OPUs show the amorphous structure as per the XRD patterns. The amorphous structure of OPUs is due to the presence of a rigid naphthyl ring that limits the movement of the polyurethane chains, leading to difficulties in crystallization [22].

It is also noted that the diffraction patterns of all samples present a weak shoulder appearing at 43–45°, indicating a reduced degree of orientation structure, which probably results from some minor level of the hard segment and BINOL [24]. It is evident that the shoulder is more clearly in the OPUs with higher BINOL content.

### 2.6. Mechanical Properties

**Figure 5:**
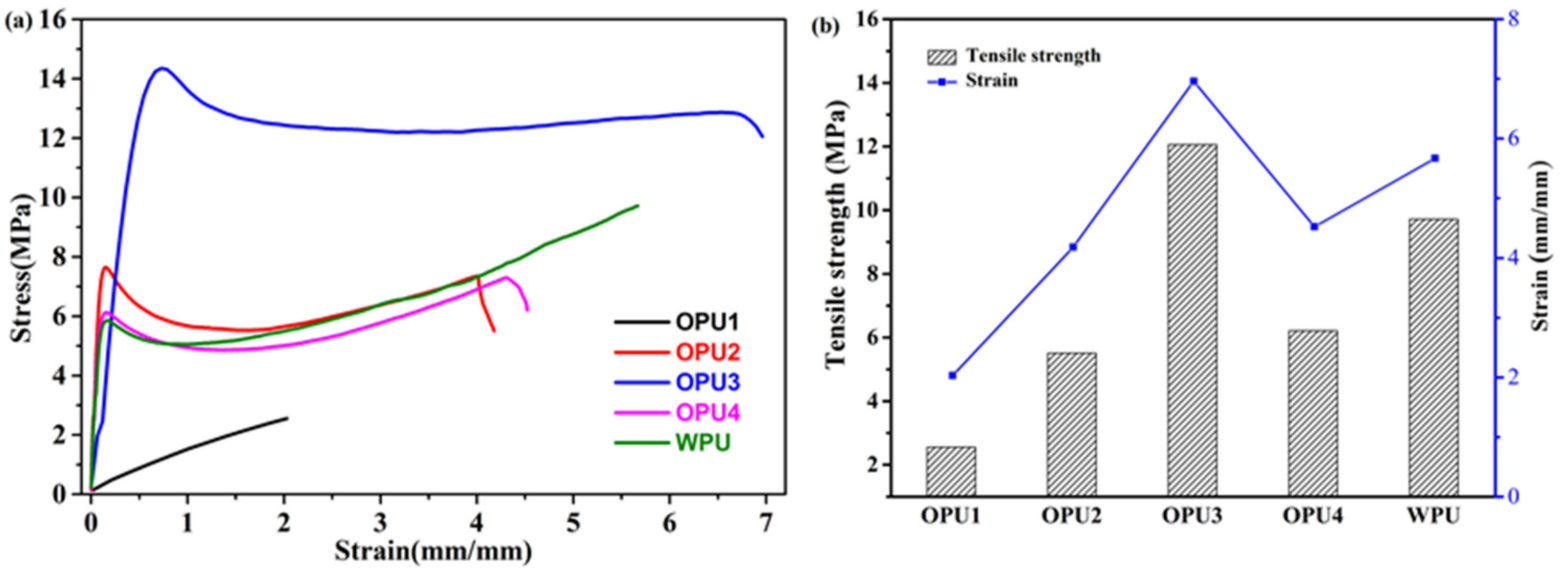
Stress–strain curves of WPU and OPUs (**a**) and the extracted values of tensile strength and stress (**b**).

The tensile testing of all samples was performed to investigate the effect of BINOL content on the mechanical properties of OPUs, as shown in the stress–strain curves in Figure 5a. The stress–strain curves demonstrate a distinct variation in tensile strength and elongation at break among these polyurethanes. OPU1 shows a typical brittle fracture behavior without any obvious yield before break. It has a low tensile strength (2.56 MPa) and less elasticity (203%). As BINOL content decreases, the tensile strength and strain increase distinctly (OPU2 and OPU3), indicating the two samples have better mechanical strength and stretchability. OPU3 with proportional BINOL and BDO exhibits the highest tensile strength at 12.06 MPa and a percent elongation before fracturing at 696%. Further decreasing BINOL content, the tensile strength, and strain decrease. Compared with WPU, OPU4 presents relatively lower stress and strain at 6.21 MPa and 452%, respectively.

It can be concluded that controlling BINOL in the synthesis process is crucial in determining the mechanical properties of OPU. The BINOL molecular shows rigid structural features. In addition, compared with the alcoholic hydroxyl group in BDO, phenolic hydroxyl groups in BINOL are less reactive, which may lead to the low molecular weight of OPUs. Thus, the resulting polyurethanes (especially OPU3) have higher tensile strength and stretchability with the increase of BINOL in OPU (from OPU4 to OPU3). This is probably due to the moderate content of BINOL and relatively high molecular weight. However, further increase of BINOL (from OPU2 to OPU1) has resulted in a significant drop in mechanical strength and stretchability due to the rigidity of BINOL and low molecular weight.

### 2.7. Infrared Emitting Ability

An object with a low infrared emissivity usually cannot be detected by an infrared detector, which can indirectly demonstrate the low surface temperature of the object. The infrared emissivity values of the samples with the wavelength range of 8–14 μm at room temperature are listed in Table 2. WPU shows a relatively high infrared emissivity value of 0.866 due to its strong absorbability at the infrared wave band as a result of high unsaturated groups in its structure. However, the infrared emissivity of OPUs was distinctly lower than that of WPU, as inferred from Table 2. In addition, the infrared emissivity decreases from 0.850 to 0.572 as the content of the BINOL monomer increases. Due to the introduction of the BINOL monomer, the orderly secondary structure of the polyurethane chain is obtained, leading to the formation of massive intermolecular interactions. As a result, the index of hydrogen deficiency and the unsaturated degree reduced [25]. It is evident that optically active polyurethanes have lower infrared emissivity values than WPU, and the infrared emissivity decreases with the increase of the content of the BINOL monomer. In addition, the infrared emissivity of the OPUs is similar to or even lower than the results in the previous study [26].

### 2.8. Infrared Thermography Analysis

**Figure 6:**
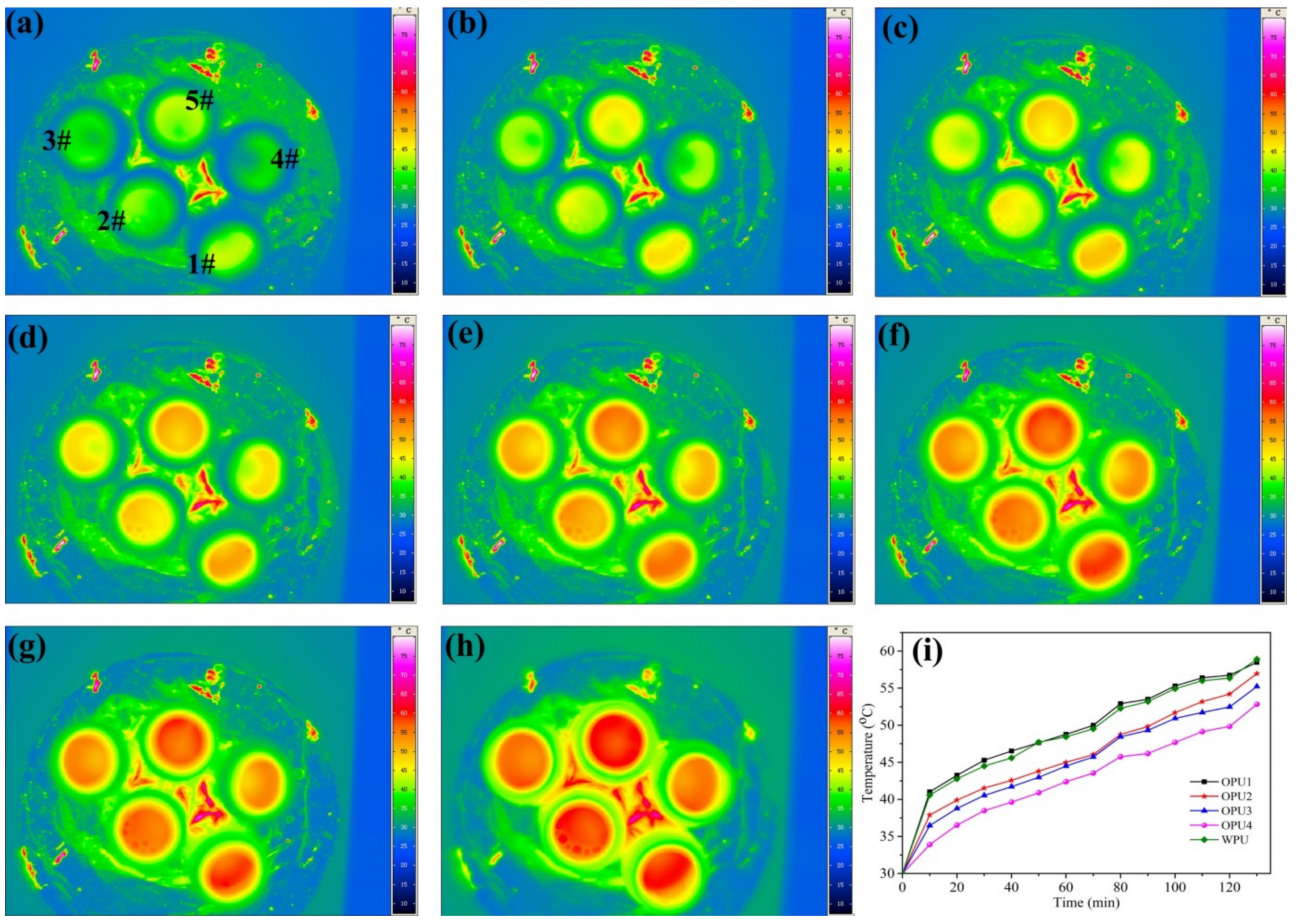
Infrared thermography of WPU and OPUs: (**a**) 0 min, (**b**) 20 min, (**c**) 40 min, (**d**) 60 min, (**e**) 80 min, (**f**) 100 min, (**g**) 120 min, (**h**) 140 min, (**i**) The variation of the surface temperature with time.

Infrared thermography can intuitively reflect the temperature field of an object, which has been used to detect the heat-shielding performance of materials. Figure 6a–h shows the infrared thermography of WPU and OPUs, in which the samples were heated from 30 °C to 60 °C, and their infrared thermal imaging was recorded in every ten min interval. It is observed that as the BINOL content decreases, the average surface temperature decreases evidently, indicating enhanced heat-shielding performance.

The surface temperature was extracted from the infrared thermographs at different time intervals to plot the surface temperature versus time curves for WPU and OPUs. As shown in Figure 6i, OPU4 exhibits the best heat insulation and cooling ability with about a 7 °C temperature difference, while OPU1 with the highest BINOL content shows relatively poor heat-shielding performance similar to WPU. This is because OPU4 with both BINOL and BDO possesses massive intermolecular interactions due to its orderly secondary structure and hydrogen bonds. The infrared thermography analysis shows that OPUs has enhanced heat-shielding performance, and the effect is comparable with that of WPU/ATO nanocomposite coatings [12,27]. These results indicate the OPUs have a great potential in thermal insulation materials.

## 3. Materials and Methods

### 3.1. Materials

Isophorone diisocyanate (IPDI), 1,1′-Binaphthalene-2,2′-diol (BINOL), and 2,2-Dimethylol propionic acid (DMPA) were purchased from Aladdin Co., Ltd. (Shanghai, China). Polycarbonate diol 1000 (PCDL1000) was available from Jining Huakai Resin Co., Ltd. (Jining, China) and was vacuum dried at 60 °C for about 24 h prior to use. In addition, 1,4-Butanediol (BDO) and triethylamine (TEA) were procured from Sinopharm Chemical Reagent Co., Ltd. (Shanghai, China). Anhydrous tetrahydrofuran (THF) was bought from Sinopharm Chemical Reagent Co., Ltd. and always kept with 4A molecular sieve before use.

### 3.2. Preparation of OPUs

**Scheme 1:**
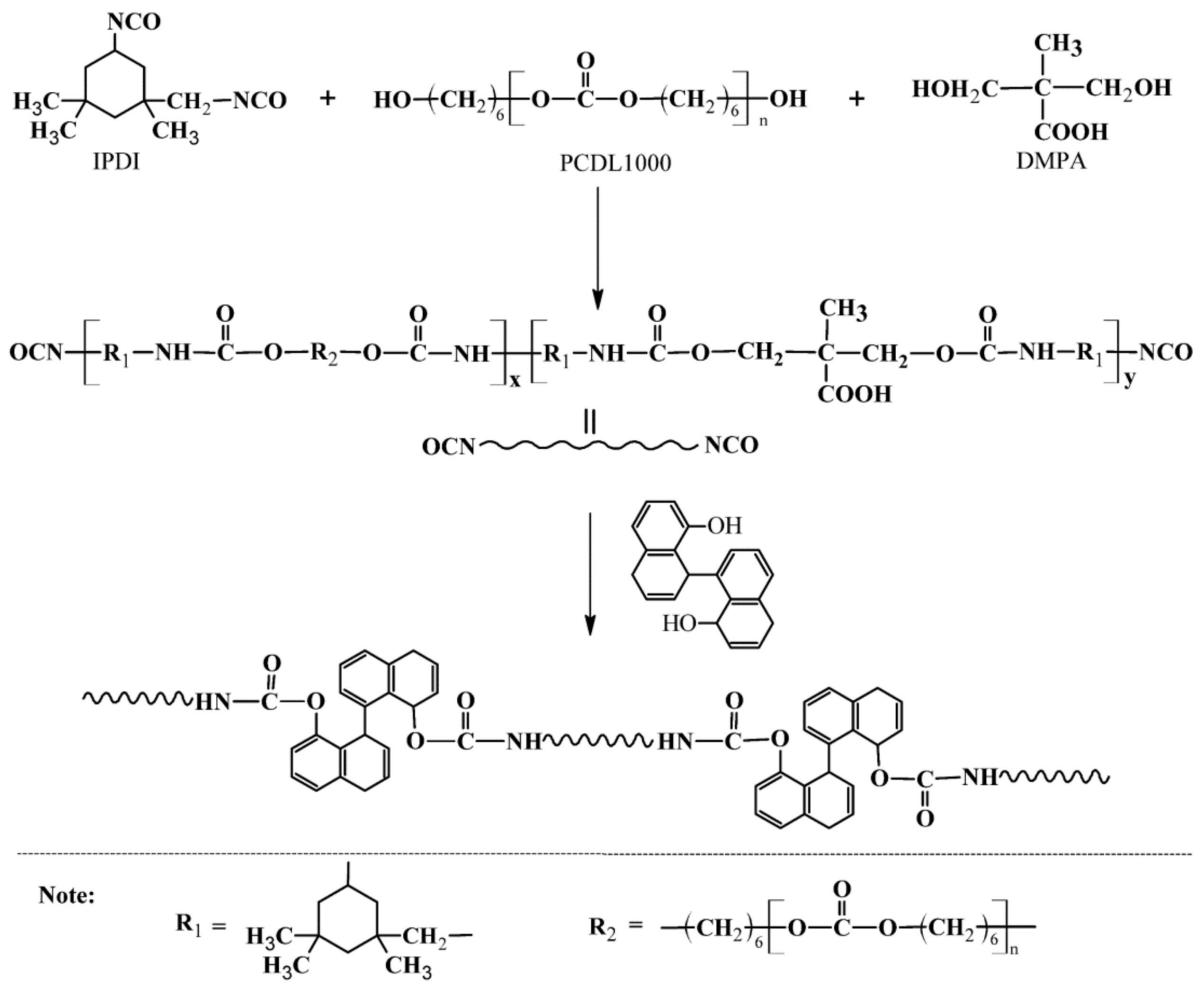
Preparation route of OPUs.

The preparation route for OPUs is shown in Scheme 1, and the molar ratio of each component is listed in Table 3.

**Figure 7:**
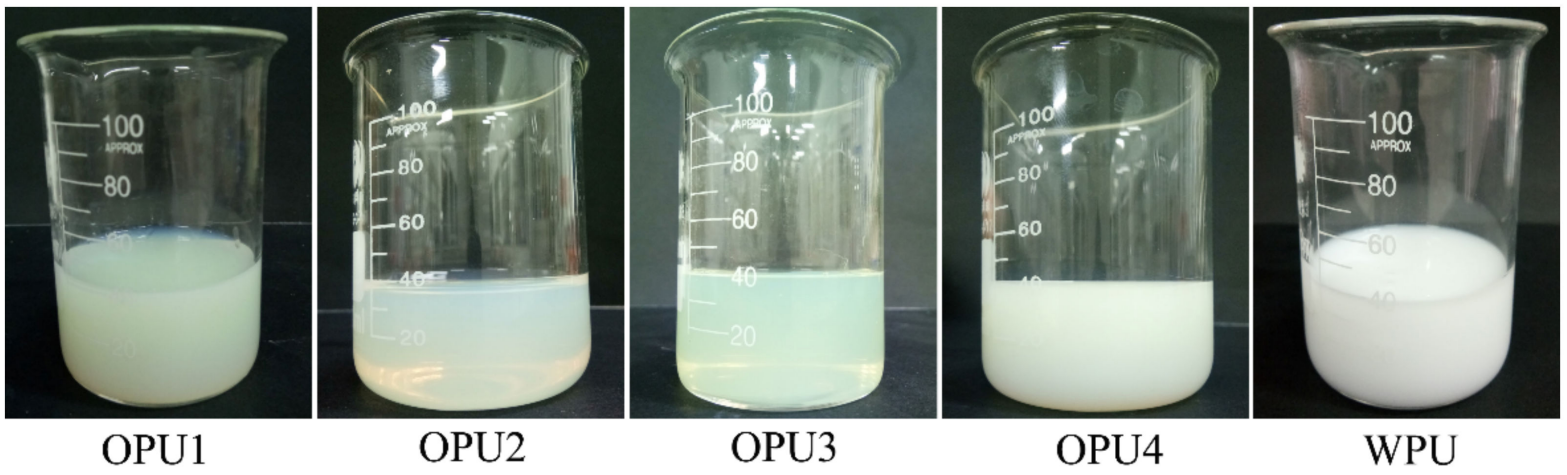
Images of OPU emulsions.

IPDI and PCDL1000 were simultaneously added into a three-necked flask equipped with a mechanical stirrer and a reflux condenser to perform prepolymerization at 75 °C for 1.5 h. A desired amount of DMPA was then added to react with the prepolymer for chain extending at 75 °C for 1.5 h. One drop of catalyst dibutyltin dilaurate was added for catalyzing the chain extension reaction BINOL dissolved in 40 mL anhydrous THF was added drop by drop into the system, and the reaction was continued at 75 °C for another 3 h. Afterward, TEA was added to neutralize carboxyl groups in DMPA for 0.5 h when the reaction system was cooled to 40–50 °C. Then, 40 mL deionized water was added drop by drop with stirring vigorously, and THF was removed via vacuum distillation to obtain OPU emulsions (Figure 7). In addition, a control sample, named WPU, was also synthesized using 1,4-butanediol instead of BINOL during the synthesis process.

### 3.3. Characterization and Measurements

The FTIR spectra of OPUs were obtained on a transform infrared instrument (STD-11202426D) in the wave number range of 500–4000 cm^−1^ via ATR accessory at room temperature.

^1^H NMR spectra were collected from a Bruker Aduance III 400 MHZ NMR instrument with CDCl3 as solvent and tetramethylsilane (TMS) as an internal standard.

The molecular weight of OPUs was determined by gel permeation chromatography (GPC) (Waters 1515 Isocratic HPLC, USA), using polystyrene (PS) as standards and tetrahydrofuran (THF) as eluent.

Thermogravimetric analysis (TGA) of OPUs was performed in a nitrogen atmosphere by a thermogravimetric analyzer (TG/DTA7300). The samples with 5–10 mg in weight were heated from 25 °C to 600 °C at a heating rate of 10 °C/min.

The crystalline structure of OPUs was detected by X-ray diffraction (XRD, D/max 2550, Rigaku, Japan) with Cu K radiation (λ = 0.1542 nm). The samples were scanned in the range of diffraction angles from 5° to 80°, with a scanning rate of 0.02°/min at ambient temperature and humidity.

The mechanical properties were presented on a material testing machine named RGD-5B, with a tensile speed of 2 mm/min at ambient temperature, according to the American Society for Testing and Materials (ASTM D5035). The tested samples were accurately prepared into dumbbell-shaped bar, with a dimension of 10 cm × 3 cm.

Infrared emitting ability was measured on an emissivity measuring instrument (IR-II, China) in 0–20 μm at 40 °C.

Heat-shielding performance was characterized by infrared thermography analysis with an infrared imaging device (varioCAM hr head, Germany). The OPU samples were dissolved in THF, which was then poured into the sample bottle and placed on the platform. During the test, the sample bottle was exposed to the thermal imager. The distance between the samples and the infrared lens was 2 m.

## 4. Conclusions

Optically active polyurethane was successfully synthesized from rotatory binaphthol monomer and polyurethane prepolymer. FTIR spectra and 1H NMR spectra indicated that BINOL has been successfully incorporated into the polyurethane chain. OPUs presented a lower molecular weight with a lower polydispersity index, as compared with that of WPU. Even though OPUs showed amorphous structure, they showed a more evident characteristic peak than WPU in XRD patterns as the naphthyl ring in BINOL consolidates the ordered arrangement of polymer chains. The OPU with BIMOL and BDO at mole ratio 1:1 presented better thermal stability. In addition, OPUs showed higher stretchability and higher tensile strength with the increase of BINOL content due to its rigid structural features and high molecular weight. Compared with WPU, the optically active polyurethanes showed lower infrared emissivity values, and the infrared emissivity decreased from 0.850 to 0.572 as the content of the BINOL monomer increased. Moreover, among all OPU samples, OPU4 showed about a 7 °C temperature difference in infrared thermography, indicating good heat-insulating effect. Based on their low infrared emissivity values and good thermal insulation effect, the obtained optically active polyurethanes can provide a new strategy to prepare efficient insulation materials and be widely applied as heat insulation coating in protective clothing, architecture, automobile, etc.

## Figures and Tables

**Table 1:** Molecular weight of WPU and OPUs.

Sample	*M_n_*	*M_w_*	PDI
OPU1	15,960	28,131	1.76
OPU3	18338	48059	2.62
WPU	19,444	40,971	2.11

**Table 2:** Optical rotation of WPU and OPUs.

Samples	Right (°)	Left (°)	Infrared Emissivity
OPU1	+73.70	−108.55	0.572
OPU2	+69.80	−107.47	0.602
OPU3	+68.13	−108.92	0.688
OPU4	+71.72	−106.65	0.850
WPU	+69.33	−107.12	0.866

**Table 3:** Molar ratio of each component for WPU and OPUs.

Code	IPDI	PCDL	DMPA	BINOL	BDO	TEA
OPU1	5	1.5	1.5	2	0	1.5
OPU2	5	1.5	1.5	1.5	0.5	1.5
OPU3	5	1.5	1.5	1	1	1.5
OPU4	5	1.5	1.5	0.5	1.5	1.5
WPU	5	1.5	1.5	0	2	1.5

## Data Availability

No new data were created or analyzed in this study. Data sharing is not applicable to this article.

## Sample Availability

Samples of the compounds are available from the authors.
